# Searching for diagnostic certainty, governing risk: Patients' ambivalent experiences of medical testing

**DOI:** 10.1111/1467-9566.13391

**Published:** 2021-10-29

**Authors:** Kiran Pienaar, Alan Petersen

**Affiliations:** ^1^ Sociology Department School of Social Sciences and Humanities Deakin University Geelong Vic. Australia; ^2^ Sociology Program School of Social Sciences Monash University Clayton Vic. Australia

**Keywords:** ambivalence, artificial intelligence (AI), Australia, biopolitics, COVID‐19, diagnostic testing, qualitative study, sick role, subjectivity

## Abstract

Diagnosis is pivotal to medicine's epistemic system: it serves to explain individual symptoms, classify them into recognizable conditions and determine their prognosis and treatment. Medical tests, or investigative procedures for detecting and monitoring disease, play a central role in diagnosis. While testing promises diagnostic certainty or a definitive risk assessment, it often produces uncertainties and new questions which call for yet further tests. In short, testing, regardless of its specific application, is imbued with meaning and emotionally fraught. In this article, we explore individuals' ambivalent experiences of testing as they search for diagnostic certainty, and the anxieties and frustrations of those for whom it remains elusive. Combining insights from sociological work on ambivalence and the biopolitics of health, and drawing on qualitative interviews with Australian healthcare recipients who have undergone testing in the context of clinical practice, we argue that these experiences are explicable in light of the contradictory impulses and tensions associated with what we term ‘bio‐subjectification’. We consider the implications of our analysis in light of the development of new tests that produce ever finer delineations between healthy and diseased populations, concluding that their use will likely multiply uncertainties and heighten rather than lessen anxieties.

**Abbreviations**
AI: artificial intelligence
CT: computed tomography

## INTRODUCTION AND BACKGROUND

As medicine's primary classificatory tool, diagnosis plays a central role in medical practice: it serves to explain individual symptoms, classify them into recognizable conditions and determine prognosis and treatment (Jutel, 2011a). It also grants access to the ‘sick role’ with its attendant rights and obligations (Parsons, 1975). Importantly, as sociologists have long argued, the process of diagnosis does not simply describe a pre‐existing condition, it helps to shape it. As medical sociologist Owen Whooley puts it, medicine's diagnostic framework endows it with the authority to ‘define the real’ (2013: 20). Over recent decades, medical testing has become an increasingly central aspect of diagnostic processes. Medical tests promise to deliver a definitive assessment of risk or diagnosis in the form of evidence based on standardized, objective measures, which translate physiological processes into mathematical terms (Leder, 1990). While there is no single agreed definition of ‘medical test’, it is the general term used to describe an array of investigative procedures used in health care, including blood tests (e.g. faecal occult blood tests, cholesterol tests, full blood counts), imaging tests (e.g. mammographies, ultrasounds, bone density scans) and biopsies (Key, 2014). Far from being objective tools, testing technologies are freighted with meaning and invested with hopes—for certainty and future good health—and are the source of social anxieties since they underpin therapeutic decisions (Gardner, 2014; Jutel, 2011b). Studies have found that for patients the fear and uncertainty inherent in diagnostic procedures are often more distressing than the diagnosis itself (e.g. Dean, 2016; Poole, 1997). Given this, it should not be surprising that tests often do not deliver the therapeutic benefits or diagnostic certainty they promise. More than simply increasing uncertainty, when testing does not provide the anticipated outcomes, it effectively places affected individuals in a liminal state between health and disease—what Timmermans and Buchbinder (2010) designate as ‘patients‐in‐waiting’.

In this article we explore individuals' ambivalent experiences of testing, drawing on interview data from an Australian study of testing in the context of clinical practice. Combining ideas from sociological research on ambivalence and on the biopolitics of health, we argue that these experiences are explicable in light of the contradictory impulses and tensions associated with what we term ‘bio‐subjectification’. In recent years, sociologists have explored the manifestations and implications of ambivalence in the contexts of health, medicine and healthcare. While in psychology, ambivalence refers to the experience of holding conflicting beliefs, views or ‘mixed feelings’ (e.g. Armitage & Conner, 2000), in sociology ‘ambivalence’ designates not just the experience of mixed emotions, but a *social* phenomenon whose expression varies across contexts and social groups (e.g. Kerr & Franklin, 2006). In an early contribution to the sociology of ambivalence, Merton and Barber conceptualized ambivalence as ‘incompatible normative expectations incorporated in a single role of a single social status (e.g. the therapist role of the physician as distinct from other roles of his or her status as researcher, administrator, professional colleague, participant in the professional association, etc.)’ (1976: 6). More recently, Zigmunt Bauman explored the dimensions and implications of ambivalence in his analysis of modernity. As Bauman argued, modernity's drive to order, to divide and classify in both thought and practice, is troubled by ambivalence; namely ‘the possibility of assigning an object or event to more than one category’ (1991: 1). He describes modernity's ‘war against ambivalence’, which is ‘identified as chaos and lack of control, and hereby frightening and marked with extension’. However, in Bauman's view, both order and ambivalence are products of modern practice, being subject to the same logic (1991: 14–15).

The concept of ambivalence has been productively applied by health sociologists to explore the tensions and complexities of diagnosis both from the perspective of health professionals (Whooley, 2010) and patients (Rhodes et al., 1999; Zarhin, 2015). Especially relevant to the focus of this article is Nettleton's (2006) analysis of patients' experiences of medically unexplained symptoms. Drawing on Bauman's conceptualization of ambivalence and interviews with neurology patients with unexplained symptoms, she suggests that, despite promising certainty, diagnostic processes oriented towards problem‐solving and classification actually *produce* even more uncertainty and anxiety. This paradoxical phenomenon, she argues, is shaped by the conditions of late modernity, specifically the rise of risk, insecurity and anxiety. This insight is instructive for our analysis as it highlights the social conditions that shape ambivalent experiences of health and illness. For those undergoing medical testing, we propose that ambivalence arises as a consequence of the conflicting expectations and demands posed by what we term ‘bio‐subjectification’: the process that produces subjects whose identities are defined in biological terms.

Bio‐subjectification is an aspect of contemporary biopolitics which involves the use of an ever‐expanding array of technologies (Rose & Novas, 2005: 450). These technologies encompass devices, tests and treatments of various kinds that facilitate individual self‐surveillance and management of health and risk in keeping with ascribed ideals of responsible citizenship. On this view, testing technologies can be understood as a modality of biopower in that they involve the use of disciplinary techniques to control and govern biological life both at the level of the individual body and the population (Rose, 2007; Rose & Novas, 2005). In proposing the concept of bio‐subjectification, we suggest that biopolitical processes not only govern ‘life itself’ but also construct normative subjectivities defined in biological and somatic terms. Our conceptualization of bio‐subjectification resonates with the notion of ‘biopolitical subjectification’ proposed by Puumeister (2019) in an account of the semiotic dimensions of ‘biopolitics’, specifically the ways in which biopolitics constitutes subjectivities. However, where Puumeister theorizes the semiotics of biopolitics, we focus on its material effects as they play out in relation to patients' experiences of medical testing, drawing attention to the double‐edged character of testing technologies as vehicles of both hope and anxiety.

Those who attend a clinic or hospital come with hopes, expectations and fears that are shaped by previous experiences of healthcare, knowledge gained from others and exposure to news and other media. As medical sociologist Annemarie Jutel comments, individuals are unlikely to consult a medical professional unless their story has ‘diagnosis as a frame, yet […] by appropriating diagnosis, [they] step outside their authorized realm of operation’. She adds, ‘They must simultaneously believe in diagnosis but leave it in the hands of the medical professional’ (2019: 58). However, the diagnostic certainty promised by testing is often elusive and involves disclosures that may be confronting and, we note, may heighten rather than lessen anxieties and uncertainties. In some cases, individuals may prefer that medical professionals withhold diagnosis since it carries both clinical and moral significance, for example, the potential for stigmatization (Jutel, 2019). Doctors, for their part, are compelled to exercise discretion in deciding what and how much to tell patients when communicating test results. In so far as testing technologies are implicated in these complex diagnostic decisions, they can be seen as mediators of both hope and anxiety. It is these tensions and the ambivalent experiences they generate that are the focus of our analysis. In exploring the complexities of these experiences, our study contributes to a growing literature in health sociology on ambivalence surrounding health technologies and diagnostic processes (e.g. Gaspar et al., 2021; Nettleton, 2006; Zarhin, 2015). Specifically, it advances our understanding of the emotional dissonance, or feeling of unease, that patients experience in relation to medical technologies in which their hopes are invested but which often fall short of their promise. We argue that these experiences are explicable in light of the contradictory impulses associated with bio‐subjectification, the ways in which particular biomedical practices and technologies constitute subjects.

## METHODS

Our empirical data comprise 34 in‐depth, qualitative interviews with healthcare recipients from diverse backgrounds, living in cities and regional areas across Australia. The data are available on request from the corresponding author; they are not publicly available due to ethical restrictions. The interviews were conducted as part of a larger sociological study of testing in Australian healthcare both in the contexts of routine clinical practice and population‐based cancer screening. This study, which was undertaken between 2017 and 2020, aimed to examine the extent to which optimistic expectations shape the practices of testing in healthcare in Australia, focusing on clinical practice and the national cancer screening programmes for bowel, breast and cervical cancer. The study comprised a number of complementary components, the findings of which have been published elsewhere including a content analysis of relevant policy documents (Pienaar et al., 2019); surveys and interviews with stakeholders in the field of testing technologies; interviews with GPs and patients about testing in clinical contexts (Pienaar et al., 2021); and interviews with participants in national screening programmes for breast, bowel and cervical cancer. In this article we focus on the experiences of patients in clinical contexts and the biopolitical implications of testing for diagnosis and risk assessment.

The selection of our sample of tests was guided by an analysis of Australian government statistics showing a steep rise in the use of certain tests in healthcare between 2003–2004 and 2014–2015 (e.g. Medical Benefits Review Task Force, 2012; Medical Benefits Schedule Review Taskforce, 2015). This included tests used for purposes of risk assessment, diagnosis, disease monitoring, ruling out underlying conditions and preventive screening. In our recruitment advertisements we called for people who had undergone, or considered undergoing, at least one of the following types of medical tests:

- Test for vitamin D or vitamin B12 deficiency;
- An imaging test for one or more conditions, for example, ultrasound, X‐ray, mammogram, CT scan (excluding tests for pregnancy);
- A genetic test for one or more conditions (excluding tests for pregnancy); and/or
- A cancer screening test (e.g. for bowel, breast or cervical cancer).

Participants were recruited between April 2018 and March 2019 by publicizing a call for volunteers on the study's website and social media, through professional networks and via a short online survey in which respondents were given the option to take part in a follow‐up interview. The study was approved by Monash University Human Research Ethics Committee (Approval number 12274). All participants provided informed written or oral consent. Interviews were conducted over the course of 11 months by the first author and a research assistant. They were undertaken telephonically to maximize geographic variation and cost‐efficiency. Interviews lasted between 30 and 125 minutes, with an average of 57 minutes. They were semi‐structured and explored experiences with diagnostic testing and screening, including what prompted participants to undergo it, the possible influence of test outcomes on subsequent healthcare decisions, perceptions of the testing process and/or results, views on risks and benefits, and informational needs and preferences regarding medical testing. Interviews were audio recorded, professionally transcribed verbatim and the transcripts were checked for accuracy. To protect participant identities, each was given a pseudonym and all identifying details were removed from the transcripts. The 34 participants comprised 27 women and 7 men, aged between 24 and 72 years with most in their 60s. Most participants were highly educated with 82% having completed a tertiary qualification.

The data were coded by the first author using an iterative inductive approach in which a preliminary list of codes was drawn up based on the study's overarching interest in the sociocultural factors shaping testing and diagnosis, knowledge of debates in the field and the relevant literatures on diagnostic testing and screening. These codes were tested on a subset of data, and the coding frame was then refined: supplementary codes were added across the data set to capture a wider range of themes derived from the interviews themselves. Saturation was reached after this broader coding frame was applied to the data. Coding was undertaken in NVivo to enable the research team to comment on emerging themes, and cross‐check for coding consistency, deviant cases and alternative interpretations (Dicks, 2012). Codes relating to experiences of testing, perceptions of the testing process and practices of managing health were reviewed in terms of how accounts of testing depict its risks and benefits, and the relationship of testing practices to diagnosis, risk governance and the broader project of healthy living.

### ANALYSIS

### The performativity of testing: Producing certainty, relief and reassurance

One of the central tenets of Western biomedicine is that testing, whether involving an assessment of tissue or blood samples, images or other techniques, can provide the potential means to diagnostic certainty, or an accurate explanation for a patient's health problem. While in practice, such certainty is very often elusive, the introduction into healthcare of increasingly sophisticated techniques of testing reflects a belief that it is ultimately achievable. In contemporary society, the quest for diagnostic certainty is seen in the ‘Dr Google phenomenon’ (Jutel, 2019: 47) where patients may self‐diagnose on the basis of an Internet search prior to, or sometimes even instead of, consulting a health professional.

When asked about the benefits of testing, a common theme in participants' responses was its role in securing a diagnosis, and consequent relief in having a diagnostic label for their symptoms (often following multiple rounds of testing). Sixty‐eight‐year‐old Beth captures this succinctly in relation to being diagnosed with Crohn's disease after months of experiencing chronic diarrhoea and undergoing various imaging tests before finally having a double balloon enteroscopy (an endoscopic technique using a flexible scope to examine the small intestine) that led to the diagnosis:

“[Testing] gave me a diagnosis, and that was a big relief. Actually putting a name to what was the problem.”

Beth (68, Western Australia)

Like Beth, Kirsten had undergone a gamut of imaging tests with no clear diagnostic outcome and describes the sense of relief that accompanied her eventual clinical diagnosis of rheumatoid arthritis. Kirsten received the diagnosis after being referred to a rheumatologist who ordered blood and imaging tests, including an MRI of her hand, which provided the basis for the diagnosis.

“I was really happy to have a diagnosis […] the overwhelming thing was that I felt like there must be some treatment now. Now that we certainly know what's going on, I felt like that was the positive thing […] I was just a bit relieved that finally there was a name for what was going on […] I was just hopeful that there would be some treatment that would work […] Along with the diagnosis [the doctor] made suggestions about what I should start taking to start to control what I was experiencing. So that was good, that was positive too.”

Kirsten (60, Victoria)

In addition to the relief associated with ‘finally [having] a name’ for her symptoms, Kirsten felt hopeful that the certainty of a firm diagnosis would lead to effective management and treatment of her condition. The striving for diagnostic certainty (‘putting a name to it’) is a common theme in the literature and can be traced back many decades. For example, in her recent book on the sociology of diagnosis, Jutel (2019) quotes Balint's 1960s work where he notes:

“The request for the name for the illness, for a diagnosis is the most pressing problem for the patient. It is only in the second instance that the patient asks for therapy… finding ‘nothing wrong’ is no answer to the patient's most burning demand for a demand for a name for his [sic] illness.”(Balint, 1964 cited in Jutel, 2019: 47).

Kirsten's account above is consistent with the sociology of diagnosis literature in that it highlights the performativity (or reality‐producing effects) of diagnosis: it is important for explaining and legitimating illness, and facilitating access to appropriate treatment (Greco, 2012). Extending this literature, we suggest diagnosis also produces bio‐subjectification in that it constitutes the patient as capable of managing their illness. In Kirsten's terms, it enabled her to take ‘control [of what she] was experiencing’.

Echoing Kirsten's views, another participant Hayden describes his palpable relief after undergoing a series of pathology and imaging tests when he eventually received a diagnosis of ulcerative colitis, which meant his persistent, debilitating digestive issues could finally be treated:

“It's a relief to have action taken and to find out what it is. It is a relief too, to have a diagnosis then and some kind of clinical advice that is beyond the things you might have already wondered about, around like diet and stuff like that.”

Hayden (24, Victoria)

Importantly, in so far as testing facilitates diagnostic closure, it plays a crucial role in bio‐subjectification. In other words, we suggest that the practices of testing are intimately entangled with, and in many cases inseparable from, the performativity of diagnosis. It is no surprise then that testing practices generate ambivalence for patients: while testing is imbued with hopes for a conclusive diagnosis and a pathway to effective treatment, it is also freighted with anxieties and fear for the future. However, these conflicting affective dimensions are often overlooked in the clinical focus on testing technologies as providing objective measures of risk or disease.

The sense of relief associated with conclusive test results was not confined to those who received a diagnosis on the basis of diagnostic tests. Those who tested negative for a particular condition described the experience as providing a sense of comfort and reassurance of the absence of disease, as illustrated by Esther's experience of diagnostic imaging testing which revealed a non‐cancerous cyst near her ovary:

“my concern was okay, if [the cyst] is cancerous, what will the diagnosis be with regard to the […] pathology of it? There was great relief the next day when the [specialist] came to the hospital and said the test results showed it was all clear.”

Esther (54, Victoria)

These accounts of the benefits of testing are not surprising given that testing technologies are designed to aid diagnosis and provide objective measures of risk or disease. As Gardner explains in an analysis of diagnostic technologies: ‘One reason why we place so much faith in diagnostic technologies is because we think of them as providing us with an *objective* glimpse of the body and disease, free of the complexity of human emotion’ (2014: 151, original emphasis). However, as our participants' accounts testify, diagnostic testing technologies are socially embedded and, therefore, freighted with emotion: in their encounters with patients and the dynamics of clinical practice, they exceed their intended clinical function and are transformed into instruments of reassurance (Ross, 2018). The affective aspects of diagnostic testing also relate to the broader functions of diagnosis: beyond merely categorizing and labelling symptoms, a diagnosis validates the existence of a particular disease, provides structure to otherwise abstract symptoms and assigns an identity to the person diagnosed. Importantly, it legitimizes their symptoms, making them legible and credible, which works to sanction the affected person's illness (Jutel, 2011b). In this respect, diagnosis grants access to the ‘sick role’ with its associated rights and obligations (Parsons, 1975). Bio‐subjectification is achieved through transforming the affected person from an individual with symptoms to a patient with a medically legible condition.

### The uncertainties and demands of testing: Ambivalent experiences

While some participants valued testing as a means of providing diagnostic certainty, and determining treatment, others recounted mixed experiences of testing and expressed ambivalence about its benefits. Many had undergone multiple rounds of testing without receiving a definitive diagnosis and their accounts exhibit strikingly similar concerns about the downsides of testing and its far‐reaching affective impacts. For example, Kate, a long‐term migraine sufferer, had submitted herself to a barrage of imaging and blood tests to identify the cause of her recurrent migraines, rule out any underlying conditions and assess her associated risk of stroke. The results were inconclusive, and she described feeling conflicted about her experience of testing:

“I guess [my experience of testing] *is conflicted* […] On the one hand [I] viewed it as a tool to get to that answer. On the other […] it was really frustrating and an exhausting process to go through […] Logistically, it was difficult in terms of work, in terms of just organising your daily life around having to go and get another bloody test […It was] quite difficult emotionally because it exacerbated some pre‐existing anxiety that I already had […] And ultimately [it was] frustrating because [neither] round of testing really gave any definitive end […] I recognised that they were necessary in order to rule things out. I understand the process, but *you still kind of get left with a sense of futility*.”

Kate (34, New South Wales, emphases added)

Here Kate references a key promise of testing as a definitive means of diagnosis (‘a tool to get that answer’). She describes her frustration when this promise failed to materialize, leaving her with the sense that undergoing multiple tests had been a futile exercise. Her account powerfully illustrates the double‐edged character of testing and other clinical investigations: they can be sources of both hope and frustration when the certainty they promise proves elusive (Nettleton, 2006). Kate's description of the logistical implications of testing reveals the penetration of these biopolitical technologies into the processes of everyday life, testifying to biopower's ever‐greater hold on all aspects of life (Marks, 2006).

Her account also demonstrates the physical and emotional impact of successive rounds of testing: not only was this logistically challenging in terms of scheduling and ‘organizing daily life’ around the next medical appointment but it also aggravated the anxiety Kate was already experiencing. In this sense Kate's experience highlights how testing serves bio‐subjectification when the outcomes prove inconclusive: in such cases testing can heighten anxiety and frustration and generate a sense of futility and possibly even fatalism. Her account resonates with Crawford's (2004: 504) analysis of anxiety in medical culture in which he argues that the expansion of medical knowledge and technologies for managing threats to health ‘aggravate[s] the very insecurities they are designed to quell’. As Crawford comments, ‘Anxiety about health, though overdetermined, is aggravated by a medical culture compelled to identify dangers in order to control them’ (2004: 504). Within this culture, the growing expectations and optimism that accompany medical advances foster an ever‐increasing intolerance of uncertainty, even as the promise of certainty proves stubbornly elusive. The uncertainty often generated through testing and other clinical investigations can provoke or increase patient anxiety, in the process reinforcing the slippery, elusive nature of diagnosis (Nettleton, 2006; Nettleton et al., 2014).

In other cases, discrepancies between test results and other diagnostic information can introduce ambiguities into the clinical picture and generate doubt about which diagnostic source to trust. The experience of 50‐year‐old Joshua highlights some of the issues at stake in such cases. Diagnosed with Crohn's disease in his early 20s, Joshua has undergone extensive imaging and pathology tests over the years in relation to various Crohn's‐related health issues. He described how, before the Crohn's diagnosis, his long‐standing GP had queried the veracity of his health concerns, suggesting that they were psychosomatic because his test results did not match his reported symptoms:

“Before I was diagnosed, I was being seen by my GP in [Australian city] who actually delivered me as a baby. And I had him for many, many years right up until my early twenties. And he actually did a range of tests because I had symptoms before […I was eventually diagnosed]. And I went to see him one day and he said, ‘I purely believe that what you're dealing with is psychosomatic’, and I said, ‘Really! […] You know, here I am presenting to you. I've known you all my life and you're telling me that it's all in my head?’ He said, ‘No, these symptoms are not right’, so I basically took myself off and left him as a doctor.”

Joshua's experience points to the significant weighting assigned to test results in evidence‐based medicine: as a hermeneutic tool of medicine (Leder, 1990), they are seen as reliable, objective measures of the presence or absence of disease. In cases such as Joshua's where there is diagnostic uncertainty and discrepancies in the clinical picture, diverging measures must be coordinated in order to achieve diagnostic coherence (Leder, 1990). This interpretive process references an established scientific hierarchy in which ‘objective’, quantitative test results trump ‘subjective’ patient self‐reports or clinical findings (Gardner, 2014). According to Leder, the reliance on quantitative data to facilitate diagnosis is a result of ‘medicine's flight from interpretation’ and its quest for ‘purified objectivity’ to escape the biases and ambiguities of clinical judgement (1990: 21). In this context, and within the hierarchy of evidence‐based medicine, test results are assumed to be impartial and thus tend to be privileged as the final arbiter of signs and symptoms (Gardner, 2014), even when the attending doctor's knowledge of the patient might indicate a need for caution in assigning them greater value than the patient's reported symptoms. We suggest that perceptions of doctor–patient relations are also at play here: in Joshua's case, the ‘traditional’ view of the doctor as the knowledgeable authority did not leave much room for questioning his clinical assessment. In terms of the performativity of testing as a biopolitical process, inconclusive or ambiguous test results also produce multiple enactments of the condition being tested for: a yet‐to‐be‐diagnosed verifiable, physiological condition (as in Joshua's depiction) or an uncertain psychogenic condition (as in his GP's assessment). Not only do these enactments rely on a reductive mind/body dualism that reinforces the negative connotations of the ‘psychological’ in diagnostic processes (Greco, 2012) they also have implications for doctor–patient relations. Where a condition is rendered illegible through inconclusive test results, it could cast doubt on the patient's reported symptoms and lead health professionals to question their veracity. This of course risks undermining the patient's credibility and limiting their capacity to advocate for and access the care they need, which can leave them feeling voiceless and disheartened (Rhodes et al., 1999).

Thus, as previously noted, when testing does not provide the anticipated outcomes, it effectively places those affected in a liminal state between health and pathology. Without a diagnosis (made legible through a positive test result), they do not fulfil the requirements of fully fledged patients and so are effectively designated ‘patients‐in‐waiting’, subject to medical surveillance until a firm diagnosis can be made (Timmermans & Buchbinder, 2010). At worst, where the discrepancies between the different diagnostic sources cannot be resolved, those affected risk having their symptoms dismissed as exaggerated, psychogenic or even fake. In Joshua's case, the fact that his GP discounted his symptoms as psychosomatic lead him to consult a different GP who took his symptoms seriously and referred him to a specialist. Within two weeks, after conducting gastro‐intestinal imaging tests, the specialist made a diagnosis of Crohn's disease.

A number of other participants were also equivocal about the value of testing and drew attention to some of its negative consequences such as the potential to generate anxiety and heightened awareness of health risks. Diane (49, Western Australia), for example, who was diagnosed with severe osteoarthritis after undergoing a series of imaging tests, likened the fear of a positive test result to a ‘bomb waiting to explode’:

“I think [being tested] produces a bit of anxiety about, you know, ‘oh my God! What are they going to find? […] is this the one where they're going to tell me that I've got cancer or is this the one that's going to tell me something really bad's going to happen?’ So it's kind of like a bomb […] I know it's there, when's it going to explode? So it's not a great feeling.”

The bomb metaphor is evocative, being freighted with meaning about the destructive power of disease: like an explosive weapon, an undiagnosed disease lurking in the body can wreak havoc at any time without notice. Disease, then, in this militaristic framing is figured as a violent threat and the body as a potential site of battle if the threat is not contained. The appeal of testing technologies lies in their promise to detect disease early and contain its destructive power. In capturing the negative valence of disease and the fears surrounding its diagnosis, Diane's account offers a powerful testament to the sense of dread and anxiety that diagnostic testing can engender. It illustrates well Gardner et al.'s argument that ‘diagnostic procedures [including testing] do not necessarily reduce or eliminate risk: [but rather] by creating an awareness of the material body and delineating problematic areas, diagnosis can increase the sense of risk’ (2011: 849).

Similar to Diane's account, but articulating more explicit ambivalence about the impact of testing, Catherine noted:

“It's great having all these medical tests and health knowledge at our fingertips, because *you can take responsibility*. And you can actually engineer something for yourself and take control of various facets of your health practices, but on the other hand *it can also make you anxious because you then become aware of all the possibilities of what might go wrong*.”Catherine (55, Victoria, emphases added)

Catherine's account highlights the double‐edged nature of testing as a biopolitical technique: on the one hand, the knowledge generated through testing is a tool for empowering individuals to manage their health (and be ‘good’ biological citizens), but on the other it can produce anxiety due to an increased awareness of ‘what might go wrong’. In this respect, far from reducing uncertainty by providing diagnostic closure, the use of technologies designed to generate knowledge about the future can actually increase uncertainty and anxiety by confronting one with a glaring sense of the risky futures that may yet materialize (Brown & Michael, 2003).

### Testing, self‐surveillance and the will to health: Producing expert patients

One of the key aspects of contemporary forms of biopolitics is the interpellation of healthcare recipients as empowered, knowledgeable advocates for their own health and wellbeing. This depiction enjoins individuals to be informed and actively involved in decisions about their health, and it is here that testing plays a crucial role as a means of health surveillance and treatment monitoring. Some participants in our study were critical of the contemporary imperative to monitor and optimize health. For many of those with chronic conditions requiring continual medical surveillance, testing and management, the ongoing attention to health was logistically and emotionally wearing. As Kirsten explains in relation to living with rheumatoid arthritis:

“You've just got to kind of pay attention. It's a process of continuously monitoring your body and how am I feeling […] it's not like a ‘set and forget’ sort of thing […] It sits always in my mind […] it's not a good thing about this kind of a chronic disease […] the processes are exhausting, the *continuous testing is exhausting* and […] it's making sure you're not running out of your medication and, you know, I've got to get to chemist before it shuts, but I'm at work and I can't get there.”

Kirsten (60, Victoria, emphasis added)

Kirsten's account highlights the embodied labour involved in managing a chronic health condition. While the various diagnostic, corrective and surveillance technologies for managing ‘defective’ bodies are designed to minimize disease risk and improve health, they also place a considerable burden on affected individuals to organize their everyday life around processes of continual monitoring and tracking associated with contemporary biopolitical techniques. Such processes—in which testing plays a key role—work to devolve responsibility for managing health onto the self‐monitoring individual (French & Smith, 2013). This increasingly patient‐oriented healthcare system reflects the blurring of the line between expert/clinical and lay/non‐clinical spheres that is a hallmark of the contemporary biopolitical order and the creation of new expectations regarding healthy living and care of the self. The contemporary emphasis on health and wellbeing has profound subjectifying effects: no longer simply passive recipients of medical care, patients are increasingly enlisted as skilled healthcare recipients who are actively involved in decisions concerning their health. As such, they become an ‘ally of the doctor’ (Rose & Novas, 2005: 489), expected to mobilize high levels of medical literacy and expertise in monitoring and advocating for their health. As our participants' accounts demonstrate, these expectations can be experienced as onerous and anxiety inducing, particularly when accompanied by surveillance testing for managing chronic conditions.

Indeed, for some of our respondents, the medical surveillance and testing associated with having a chronic condition was a source of considerable anxiety. Thirty‐nine‐year‐old Iris has osteoporosis, Vitamin D deficiency and hyperthyroidism linked to Grave's disease. Although as she notes, none of these conditions is life‐threatening, they require her to undergo regular pathology tests to monitor their status and adjust treatment if needed:

“The worry […] I was just worried all the time […] I just felt like I'd had this constant background hum, like a low‐level anxiety about some health problem or another. Like my thyroid, my broken wrist, the osteoporosis. And they were all low‐risk problems. Like it wasn't dire, like I wasn't going to die from any of them, but all of them had this long‐term attention to my health, and intervention and treatment and monitoring and blood tests […] It was all quite annoying in the long run […] It's not good for me to be always thinking about, ‘What does this mean for me?’”

Iris (39, Victoria)

Here, Iris describes how the experience of ongoing medical surveillance prompted her to adopt a stance of vigilant introspection consistent with the normative injunctions of contemporary forms of biopolitics, which involved continually interrogating the implications of particular test results for her health. Her account suggests that far from being a neutral means of measuring risk and diagnosing disease, testing functions as a technique of self‐surveillance, reinforcing prevailing biopolitical discourses of individual responsibility, self‐care and self‐management of health risks (French & Smith, 2013). After years of operating in this vigilant risk‐oriented mode, Iris has since renounced the expectation that she engage in processes of continual self‐surveillance and self‐examination in the name of optimizing health and managing risk. As she explains in relation to her conflicted feelings about an upcoming bone density imaging test to monitor the effects of osteoporosis:

“I've got […] the next bone density check and I don't really want to know the result, because what if after all the Vitamin D and calcium supplementation and all the strength training, what if it's worse? […] I'm already sort of dreading this next scan and debating whether I say to my endocrinologist, ‘Can you just not tell me what the results are unless there's a real need to know?’ […] *I don*'*t want to be feeling more fragile again* […] I know if I hear, ‘No, the bones are much thinner, they've got even worse’ then […] I might go back to the psychologist to figure out a new plan of attack.”(emphasis added)

Iris' account gestures to the psychological impact of surveillance testing and the associated imperative to undertake intensive work on the self to halt or minimize the disease progression (e.g. through attention to diet and supplement intake, strength training and lifestyle changes). She invokes her deep‐seated fear and anxiety that despite her best efforts to minimize the effects of osteoporosis, the tests might show that her bones have thinned further which could lead to a sense of fatalism about her prognosis. Iris' anxiety is connected to the temporal dimensions of testing: as a prognostic measure, testing can be used to project futures of illness, disability or recovery. Implicit in these futures is a normative orientation towards cure or rehabilitation, the elimination or at least the minimization of impairment (Kafer, 2013). This orientation is buttressed by the modernist drive towards certainty, solutions and closure, yet as Bauman notes, in postmodern society we are living ‘under a condition of uncertainty which is permanent and irreducible’ (1997: 21). It is these irreconcilable tensions that generate ambivalence and conflicting bio‐subjectification effects for patients. In Iris' case, the ongoing testing not only provokes anxiety and possible fatalism about the progression of the disease, it has damaging subjectification effects in constituting her as ‘fragile’. For all these reasons she expresses a desire to resist being depicted as an informed, active health citizen who submits to continual practices of measurement and assessment in the name of health. Although, at the time of our study, Iris was still complying with the requirement to undergo regular bone density tests, she maintained that she would rather not know the results of her latest test unless a ‘real need’ exists. There is a sense in which by refusing the determinacy and certainty promised by test measures, Iris maintains the possibility of an open‐ended future, rather than a future mapped in terms of biomedical causality, curative temporality and the normative calculations of risk that attend it.

In summary, our study revealed the ambivalent views of healthcare recipients and patients about the value, risks and benefits of testing and other investigative procedures. While some described testing technologies as useful diagnostic tools or as providing reassurance of the absence of disease, others expressed reservations about testing, observing its potential to increase anxiety and render individuals responsible for managing health risks. Given these findings, we argue that testing, far from being a neutral means of measuring risk and diagnosing disease, operates as a mode of self‐surveillance and bio‐subjectification that is consistent with broader social changes over the last few decades, especially the emphasis on individual responsibilization in healthcare and more generally (French & Smith, 2013). We also suggest that participants' expressed ambivalence towards testing regimes points to a tension between the promise of testing to reduce uncertainty and assist diagnosis, and the fact that testing sometimes introduces further doubt and ambiguity into the clinical picture, in the process exacerbating patient anxiety. In the concluding section, we explore the implications of this tension for citizens' experiences of health and for health care in the future.

## DISCUSSION AND CONCLUSION

In healthcare, the value of testing for diagnosis, risk assessment and disease monitoring is largely unquestioned. Testing promises diagnostic certainty, of gaining a definitive label for a disease or condition, which will assist patients in deciding on treatment and/or care (Gardner, 2014). For patients themselves, this certainty carries the prospect of ontological security—of re‐establishing a sense of order and continuity in their lives—which is important to those who are grappling with the disruptions of a severe ongoing illness. Yet, as our study reveals, individuals' *feelings* about testing are often of ones of ambivalence, with hope being accompanied by fear and dread. For these individuals, the certainty and sense of security promised by testing is illusory: the promised freedom from illness receding from the horizon as each round of testing fails to provide a definitive diagnosis. Meanwhile, the commitment of time, energy and emotional work may be substantial, and prove exhausting. The subjectifying regimes of testing may reinforce a sense of fragility and uncertainty that is experienced as *disempowering*—the very opposite of what technologies of testing promise. By materializing the body as a locus of risk where the spectre of disease looms large, testing contributes to a notion of health as a provisional state that can never be taken for granted. This experience of ambivalence associated with bio‐subjectification is rarely if ever acknowledged in healthcare but, we argue, should be since the costs for patients and their families in terms of their experiences of health may be significant.

To be sure, healthcare professionals *do* recognize that unwarranted and excessive testing entails financial costs and produces potential harms resulting from unnecessary treatment. However, the locus of the 'problem’ is seen to lie with human decisions about the use of tests in particular contexts, such as in clinical practice or preventive screening. The international Choosing Wisely campaign, for example, is focused on raising awareness about the use of tests, treatments and procedures where they provide no benefit or may lead to harm, with advocates providing ‘best practice’ examples of efficient testing. This construction of the 'problem’, we suggest, diverts attention from the bio‐subjectifying implications of the diffusion of the practices of testing in healthcare. Being a ‘healthy citizen’ in contemporary society requires individuals to adopt a level of self‐vigilance involving personal demands and costs that are profound but mainly overlooked in debates about over‐testing or inappropriate testing. In exploring the complexities of individuals' ambivalent experiences of testing in healthcare, our study responds to the appeal made by Timmermans and Berg (2003) for research on the social impacts of seemingly mundane, ubiquitous medical technologies that mostly escape sociological attention. More specifically, in applying the concept of biopolitics to an analysis of individuals' experiences of testing, we address calls for empirical research to track the role of medical technologies in producing new kinds of bio‐subjectivities or technoscientific identities (Casper & Morrison, 2010; Clarke et al., 2010). Our findings advance understanding of how medical testing operates as a form of biopolitical intervention that generates new ontologies of health, risk and disease with significant implications for patients, prompting them to reconceptualize their identities in normative medical terms (Gardner, 2014).

The rapid introduction into healthcare of a growing array of new testing technologies, including genomics‐based and AI‐driven innovations, which promise earlier, more accurate, ‘personalized’ diagnoses, look set to intensify bio‐subjectification. Such innovations, which rely on the rapid analysis of large data sets (‘big data’) that may be drawn from various sources (e.g. images, genomic data, personal health records) and allow diagnoses at ‘point of care’ potentially ‘disrupt’ not just how healthcare is practised—the common discourse on big data innovations—but serve to heighten citizens' expectations of testing to an unprecedented level. The COVID‐19 pandemic has amplified these issues, increasing expectations and reliance on testing even as it has given rise to concerns about the accuracy of tests and the impacts of testing. In light of the evidence we have presented, it is doubtful that greater reliance on sophisticated technologies to aid decision‐making can ever deliver the certainty and ontological security that tests promise. Indeed, the future of healthcare, we suggest, is likely to be of increasing uncertainty and anxiety, and ultimately greater demands on healthcare systems posed by the growing number of ‘presymptomatically ill’ and ‘worried well’, whose emergence is directly linked to biopolitical imperatives to manage risk and optimize health.

## CONFLICT OF INTEREST

The authors declared no potential conflicts of interest with respect to the research, authorship and/or publication of this article.

## AUTHOR CONTRIBUTIONS

**Kiran Pienaar:** Conceptualization (lead); Data curation (lead); Formal analysis (lead); Investigation (equal); Methodology (supporting); Project administration (lead); Writing‐original draft (lead); Writing‐review & editing (lead). **Alan Petersen:** Formal analysis (supporting); Funding acquisition (lead); Investigation (equal); Methodology (lead); Project administration (supporting); Supervision (lead); Writing‐original draft (supporting); Writing‐review & editing (supporting).

## Data Availability

The data that support the findings of this study are available on request from the corresponding author. The data are not publicly available due to privacy or ethical restrictions.
